# Neutral Polysaccharides From *Hohenbuehelia serotina* With Hypoglycemic Effects in a Type 2 Diabetic Mouse Model

**DOI:** 10.3389/fphar.2022.883653

**Published:** 2022-05-05

**Authors:** Qinghong Liu, Jing Wu, Peng Wang, Yuxiao Lu, Xinhe Ban

**Affiliations:** ^1^ Department of Vegetables, College of Horticulture, China Agricultural University, Beijing, China; ^2^ Institute of Laboratory Animal Sciences, Chinese Academy of Medical Sciences and Comparative Medicine Center, Peking Union Medical College, Beijing, China; ^3^ Department of Environmental and Chemical Engineering, Tangshan College, Tangshan, China; ^4^ Zhumadian Academy of Agricultural Sciences, Zhumadian, China

**Keywords:** *Hohenbuehelia serotina*, neutral polysaccharide, structural characterization, type 2 diabetes, hypoglycemic effect

## Abstract

Neutral polysaccharides (NHSPs) from the mushroom *Hohenbuehelia* *serotina* were purified by D301/D152 resin ion-exchange chromatography and DEAE-cellulose anion exchange chromatography. The weight-average molecular weight (M_W_) and number-average molecular weight (Mn) of NHSP were 1,821 and 820.55 kDa, respectively. A monosaccharide component analysis showed that NHSP was composed of glucose, galactose, and mannose in molar ratio 2.6:2.1:1.0. FT-IR and NMR (^1^H and HSQC) spectroscopic analyses revealed that NHSP contained mainly 1,3-linked β-D-glucose, 1,4-linked β-D-glucose, 1,6-linked β-D-mannose, 1,6-linked α-D-mannose, and 1,6-linked β-D-galactose. The thermogravimetric analysis (TGA) showed that NHSP has good thermal stability below 250°C. NHSP notably reduced the blood glucose level (hypoglycemic effect) at dose 200 mg/kg for 21 days in a type 2 diabetic mouse model. NHSP reduced the liver index significantly, suggesting that it may help prevent hepatic steatosis or hepatomegaly.

## Introduction

Diabetes mellitus is a chronic metabolic disease characterized by sustained hyperglycemia. The primary causes of the disease are insulin secretion deficiency resulting from pancreatic β-cell impairment and hyposensitivity to insulin (Tfayli et al., 2009; Talchai et al., 2012). Diabetes may lead to systemic complications and premature death (Long and Dagogo-Jack, 2011). The number of diabetes cases worldwide has risen steadily in recent decades. Most hypoglycemic agents have undesirable side effects such as inducing hypoglycemia, weight gain, and dementia (Harsch et al., 2018). To find safe and novel hypoglycemic materials is becoming more important.

Mushroom polysaccharides have been studied extensively in the fields of biological chemistry and medicine. Studies during recent decades have revealed beneficial biological effects of various mushroom polysaccharides, including immunomodulatory, antitumor, antioxidant, hypoglycemic, hypolipidemic, and anti-radiation effects (Yuan et al., 1998; Hwang et al., 2005; Zhang et al., 2006; Sun et al., 2008; Bin, 2010; Zhang et al., 2011; Xiao et al., 2012; Li et al., 2019; Guo et al., 2020a; Guo et al., 2020b). These polysaccharides are generally nontoxic and do not have significant side effects when ingested.

The biological functions of mushroom polysaccharides are based on their structures. The structural characterization of polysaccharides, including molecular weight, monosaccharide composition, glycosidic bond configuration, and functional groups, is very important (Moradali et al., 2007). The immunomodulatory and antitumor effects of lentinan (from *Lentinula edodes*) are based on its main chain consisting of β-(1→3)-D-glucan (Meng et al., 2016). *Grifola frondosa* polysaccharide (composed of glucose, mannose, galactose, arabinose, xylose, and fucose) also displays antitumor activity by activating macrophages and T cells (He et al., 2017). Li et al. (2019) chelated chromium ions and *Ganoderma lucidum* polysaccharide (GLP) into the *Ganoderma lucidum* polysaccharide chromium (III) [GLP-Cr(III)] complex, which had better hypoglycemic effects than GLP. The primary sites of chromium (III) binding in GLP were O-H groups and C-O groups. For the hypoglycemic activity of mushroom polysaccharides, there is little investigation into the relationships between the structure and function. Analyzing the relationship between the function and structure is helpful to improve the application of mushroom polysaccharides in hypoglycemia.

*Hohenbuehelia serotina* is considered as one of the most delicious edible mushrooms. Investigations about the effects of *H. serotina* polysaccharides (HSPs) on antioxidant and anti-radiation activities have been going on, and some primary structures of HSPs with antioxidant activity were explored (Li et al., 2013; Li et al., 2017). However, there has been no detailed structural analysis of purified HSPs with hypoglycemic effects especially.

Here, we characterized one purified water-soluble neutral HSP (NHSP) by gel permeation chromatography (GPC), HPAEC-PAD, FT-IR, NMR (^1^H and HSQC), thermogravimetric analysis (TGA), and scanning electron microscope (SEM) analyses and investigated the hypoglycemic effect of NHSP in a type 2 diabetic mouse model.

## Materials and Methods

### Materials

Dry *H. serotina* fruiting bodies were obtained from a mushroom farm in Jilin Province, China. D301 and D152 ion exchange resins were from Anhui Wandong Resin Technology Co. DEAE-Cellulose Fast Flow, bovine serum albumin (BSA), and streptozotocin (STZ) were from Sigma-Aldrich Co. PL pullulan polysaccharide standards (peak average MW 783, 12,200, 100,000, and 1,600,000) were from Polymer Laboratories. L-arabinose, D-glucose, D-galactose, D-mannose, D-xylose, glucuronic acid, and galacturonic acid were from Dionex. Metformin hydrochloride (MET) was from Beijing Coway Pharm Co. The glucose-reagent kit was from Beijing Yicheng Biological Electronic Technology Co. All other reagents were of analytical grade, from Sinopharm Chemical Reagent Co.

### Isolation and Purification of Neutral Polysaccharides

Dry *H. serotina* fruiting bodies were ground to powder (40–60 meshes), extracted with boiling water (sample/water = 1:12 w/v) for 3 h, and centrifuged at 10,000 × g (15 min, 20°C) (Radzki et al., 2016). The supernatant was precipitated with three volumes of ethanol and stored overnight at room temperature. The precipitate, crude polysaccharides, was collected by centrifugation at 8,000 rpm for 15 min and dissolved in deionized water. Proteins in the crude polysaccharides were removed with trichloroacetic acid (TCA) and butyl alcohol. After centrifugation at 8,000 rpm for 20 min, the supernatant was dialyzed (MWCO 3,500 Da) exhaustively with deionized water for 48 h. The retained portion was a crude HSP solution without proteins.

**FIGURE 1:**
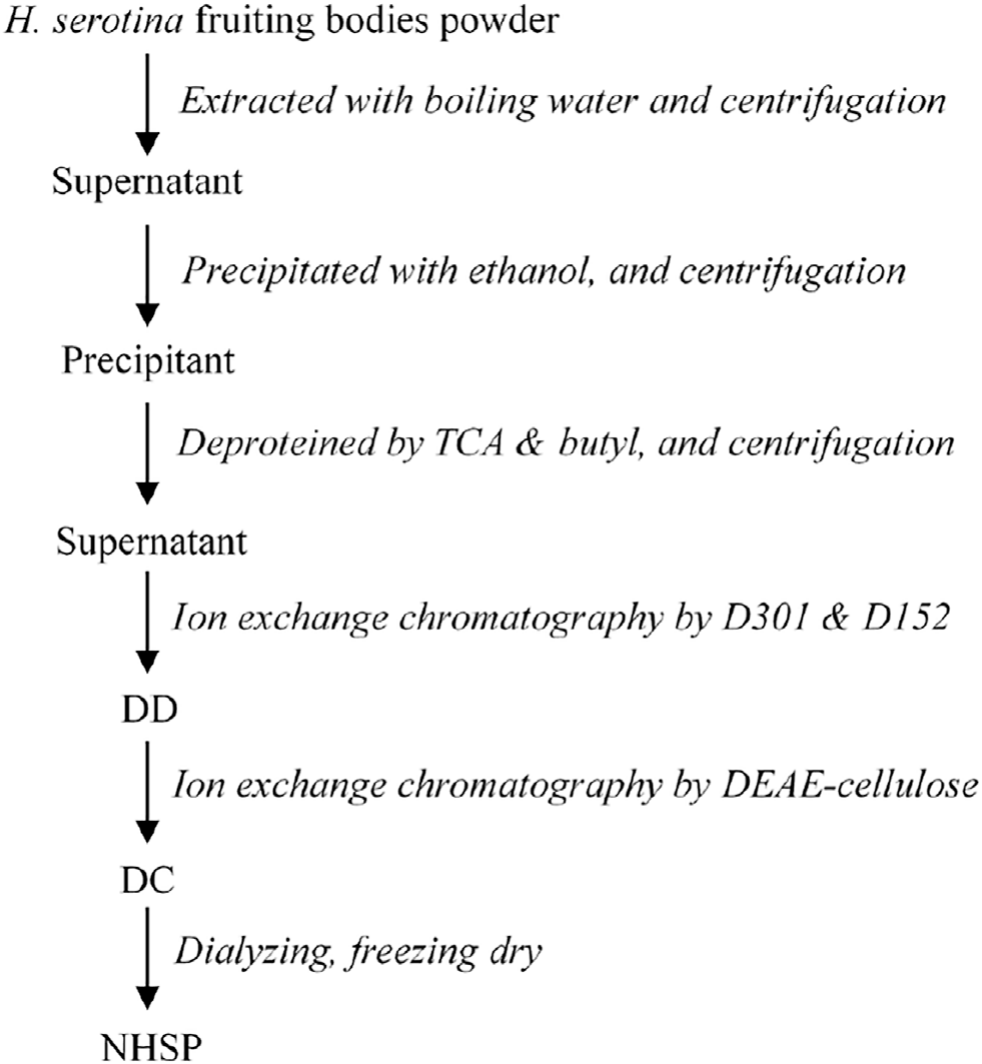
Purification scheme of *Hohenbuehelia serotina* polysaccharides.

The crude HSP solution was subjected to a D301 anion exchange resin column and D152 cation exchange resin column in series and eluted with deionized water. The unabsorbed fraction (DD) was subjected to a DEAE-Cellulose Fast Flow column and eluted stepwise in 0 mol/L and 0.5 mol/L NaCl solutions. Most of the DD was not absorbed in the DEAE-Cellulose column. This unbound fraction was collected and named DC. The DC was dialyzed with water and then precipitated with ethanol. The precipitate was lyophilized to obtain the polysaccharides termed NHSP (Figure 1).

### Carbohydrate and Protein Content

The total carbohydrate content of NHSP was determined by the phenol–sulfuric acid method using D-glucose as the standard (Dubois et al., 1956). The protein content of NHSP was measured using the Bradford method using bovine serum albumin as the standard (Bradford, 1976).

### Monosaccharide Component Analysis

The monosaccharide composition of NHSP was analyzed by high-performance anion exchange chromatography (HPAEC) coupled with a pulsed amperometric detector (PAD). Neutral sugars and uronic acids were released by hydrolysis with 10% H_2_SO_4_ for 2.5 h at 105°C. The NHSP was then diluted 50-fold, filtered, and injected into an HPAEC system (Dionex ISC 3000; United States) with an amperometric detector, AS50 autosampler, CarbopacTMPA-20 column (4 × 250 mm; Dionex), and guard PA-20 column (3 × 30 mm). Neutral sugars and uronic acids were separated in isocratic 5 mM NaOH (carbonate free and purged with nitrogen) for 20 min and then subjected to a NaAc gradient (0–75 mM) in 5 mM NaOH for 15 min. The columns were washed with 200 mM NaOH for 10 min to remove carbonate and eluted for 5 min with 5 mM NaOH for re-equilibration before the next injection. The total analysis time was 50 min and the flow rate was 0.4 ml/min (Bian et al., 2012). Calibration was performed using standard solutions of L-arabinose, D-xylose, D-glucose, D-mannose, D-galactose, glucuronic acid, and galacturonic acid.

### Molecular Weight Determination

The molecular weight of NHSP was determined by GPC on an Agilent 1200 HPLC system equipped with a PL aqua gel-OH 50 column (7.7 × 300 mm) and differential refractive index detector (Polymer Laboratories). NHSP (5.0 mg) was dissolved in 1.0 ml buffer (0.2 mol/L Na_3_PO_4_, 0.02 mol/L NaCl, pH 7.5) and filtered through a membrane (0.22 μm). NHSP solution of 20 μL (0.1 mg NHSP) was injected in each run and eluted with buffer at a flow rate of 0.5 ml/min at 30°C. The molecular weight was calculated using a calibration equation obtained from PL pullulan polysaccharide standards.

### Fourier Transform Infrared Spectroscopy Analysis

FT-IR spectra were recorded using an FT-IR microscope (model iN10, Thermo Nicolet Corp.; Madison, WI), equipped with a liquid nitrogen-cooled MCT detector, in the range 700–4,000 cm^−1^. A dried NHSP sample (2 mg) was mixed with 200 mg KBr, ground to a diameter of 2 μm using a ball mill, then pressed in a micro compression cell made of diamond. The pressed sample was placed on the FT-IR microscope stage and analyzed with pressed KBr as the blank control.

### Nuclear Magnetic Resonance Measurements

Solution-state ^1^H-NMR and heteronuclear single quantum correlation (HSQC) spectra of the NHSP sample were recorded using a 400-MHz spectrometer (model AV-III, Bruker; Germany) at 25°C. For ^1^H-NMR measurements, a 15-mg NHSP sample was placed in a 5-mm NMR tube and dissolved in 1.0 ml D_2_O, and observed chemical shifts were calibrated relative to the signal of D_2_O at 4.7 ppm (Peng et al., 2010). HSQC spectra were acquired in a gradient-enhanced (GE) experiment mode, using a 20-mg NHSP sample dissolved in 1.0 ml D_2_O. Spectral widths were 1800 Hz for the ^1^H dimension and 10,000 Hz for the ^13^C dimension. The delay between transients was 2.6 s, and the delay for polarization transfer was set to correspond to the estimated average ^1^H-^13^C coupling constant of 150 Hz. Data were processed using the Bruker Topspin-NMR software program (Peng et al., 2010; Li et al., 2011).

### Thermogravimetric Analysis

TGA and DTG were conducted with a simultaneous thermal analyzer (STA449F3, Netzsch, Germany). Approximately 8 mg of NHSP powder was placed in a platinum crucible. Nitrogen as the purge gas was used at a flow rate of 50 ml/min. The heating rate was 10°C/min in the temperature range of 30–800°C (Rozi et al., 2019). Data were analyzed by the use of Origin 8.0.2.8.

### Scanning Electron Microscopy Analysis

The dried NHSP sample was sputter-coated with gold using a sputter-coater (IB-3, EiKo, Japan) before observation. The shape and surface characteristics of the NHSP were collected at an accelerating voltage of 10.0 kV with magnifications of ×100, ×500, ×1,000, and ×2000 using a scanning electron microscope (SEM) (S-3400N, Hitachi, Japan) under high vacuum conditions (Cui et al., 2019).

### Hypoglycemic Effects in Type 2 Diabetic Mice

Male BALB/c mice (age 6 weeks, weight 18–20 g) from Beijing HFK Bioscience Co. (SCKK 2014-0004) were acclimatized for a week before experiments. The mice were maintained under controlled conditions: temperature 25 ± 2°C, humidity 50 ± 5%, and 12 h light/12 h dark cycle.

The mice in the control group were fed a standard diet (Beijing Keaoxieli Feed Co.), and referred to be group NC. Mice in the diabetic model group were fed a high sugar/high fat diet (59% standard feed, 20% sucrose, 15% lard, 5% cholesterol, and 1% cholate) (Beijing HFK Bioscience) for 4 weeks, and then the mice were intraperitoneally injected with freshly prepared 1% STZ (40 mg/kg) in citrate buffer (pH 4.3) for 5 days (Liu et al., 2018). After 24 h of the last injection, the fasting blood glucose (FBG) was measured and stable for a week. Mice with FBG ≥11.0 mmol/L were considered a successful type 2 diabetic mice model (Wu et al., 2017).

All the mice, normal mice and type 2 diabetic mice, were divided into six groups (each *n* = 5) as follows:

Group NC: normal, normal saline (10 ml/kg, i.g., for 3 weeks).

Group MC: model, normal saline (10 ml/kg, i.g., for 3 weeks).

Group MET: model, positive, metformin (40 mg/kg, i.g., for 3 weeks).

Group NHSP (100): model, NHSP (100 mg/kg, i.g., for 3 weeks).

Group NHSP (200): model, NHSP (200 mg/kg, i.g., for 3 weeks).

Group NHSP (400): model, NHSP (400 mg/kg, i.g., for 3 weeks).

During feeding, FBG and body weight were recorded for all groups on days 0, 7, 14, and 21. At the end of the experiment, all mice were anesthetized by i.p., injection of 3% sodium pentobarbital (50 mg/kg) and then killed, and the liver, kidneys, spleen, and pancreas were removed and weighed. For each of these visceral organs, an index (mg/g) was calculated as the weight of the organ (mg) divided by the body weight (g) (Guo et al., 2013). All experiments were previously approved by the Institutional Ethics Committee of China Agricultural University (No. CAU20180420-5) and were carried out in accordance with the international standards and the ethical guidelines on animal welfare.

### Statistical Analysis

All data were analyzed using SPSS (20.0) by one-way ANOVA, and *p* < 0.05 was regarded as a significant difference. All results are presented as the means ± S.E.

## Results

### Isolation and Purification of Neutral Polysaccharides

Crude *H. serotina* polysaccharides (CHSPs) (30.62 g) were obtained from 3,000 g of dried *H. serotina* fruiting bodies by hot water extraction, with a yield of 7.02%. CHSP were subjected to deproteinization, dialysis, and D301/D152 resin ion-exchange chromatography, and the unabsorbed fraction DD was further subjected to DEAE-cellulose column chromatography. The unbound fraction DC eluted with water from the DEAE-cellulose column was collected. The fraction DC was further subjected to dialysis and lyophilization, resulting in the respective production of NHSP (white powder, 20.02 g). NHSP had high total saccharide content (97.63%) and low protein content (1.98%).

### Molecular Weights and Monosaccharide Components

The MWs of NHSP were determined by GPC. According to the calibration curve with standard pullulan, the weight-average molecular weight (M_W_) and the number-average molecular weight (Mn) of NHSP were 1,821 and 820.55 kDa, respectively. The polydispersity index (PDI) (M_W_/Mn) of NHSP was 2.21, which indicated that NHSP had high homogeneity.

According to the HPAEC-PAD analysis, NHSP were composed of glucose, galactose, and mannose in the molar ratio of 2.6: 2.1: 1.0 (Table 1). This indicated that NHSP were heteropolysaccharides, and glucose might be the major unit of the main backbone of NHSP for its highest content.

**TABLE 1:** Monosaccharide compositions of NHSP.

Sample	Monosaccharide	Concentration (mg/L)	Mass fraction (%)	Molar ratio
NHSP	Glucose	11.7508	45.67	2.6
	Galactose	9.4788	36.84	2.1
	Mannose	4.4998	17.49	1.0

### Fourier Transform Infrared Spectroscopy Analysis

**FIGURE 2:**
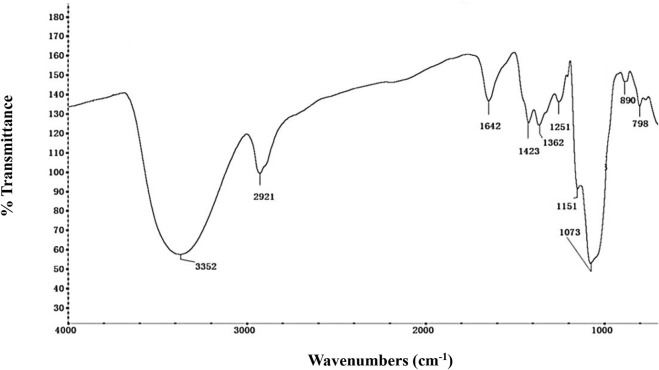
FT-IR spectra of NHSP.

The FT-IR spectrum was used to identify the NHSP based on their functional groups (Figure 2). The wide and strong peaks at 3,300–3,500 cm^−1^ and a sharp peak at 2,921 cm^−1^ were derived from O-H stretching and C-H stretching vibrations, respectively. The bands at 1,642 and 1,423 cm^−1^ were attributed to the anti-symmetric and symmetric stretching vibrations of the C=O in the carboxyl group. Specific peaks at 1,362 cm^−1^ result from an O-H variable angle vibration. The band at 1,251 cm^−1^ resulted from the C-H variable angle vibration (Zhang et al., 2014). The band at 1,151 cm^−1^ reflects C-O-C stretching vibration. Strong absorption peaks at 1,073 cm^−1^ are due to the presence of the pyranose form of a glucosyl residue (Wu et al., 2017). The absorption peaks at 890 cm^−1^ and 798 cm^−1^ indicate the co-existence of α- and β-glycosidic bonds, especially carbohydrate C1-H deformation and ring-stretching frequency are characteristics of dominant β-glycosidic linkages between sugar units (Bao et al., 2013). Glucose and mannose are dominant in NHSP, and glucose is the backbone unit which is consistent with the composition of monosaccharides in NHSP (Liu et al., 2020; Yang et al., 2020).

### Nuclear Magnetic Resonance Measurements

**FIGURE 3:**
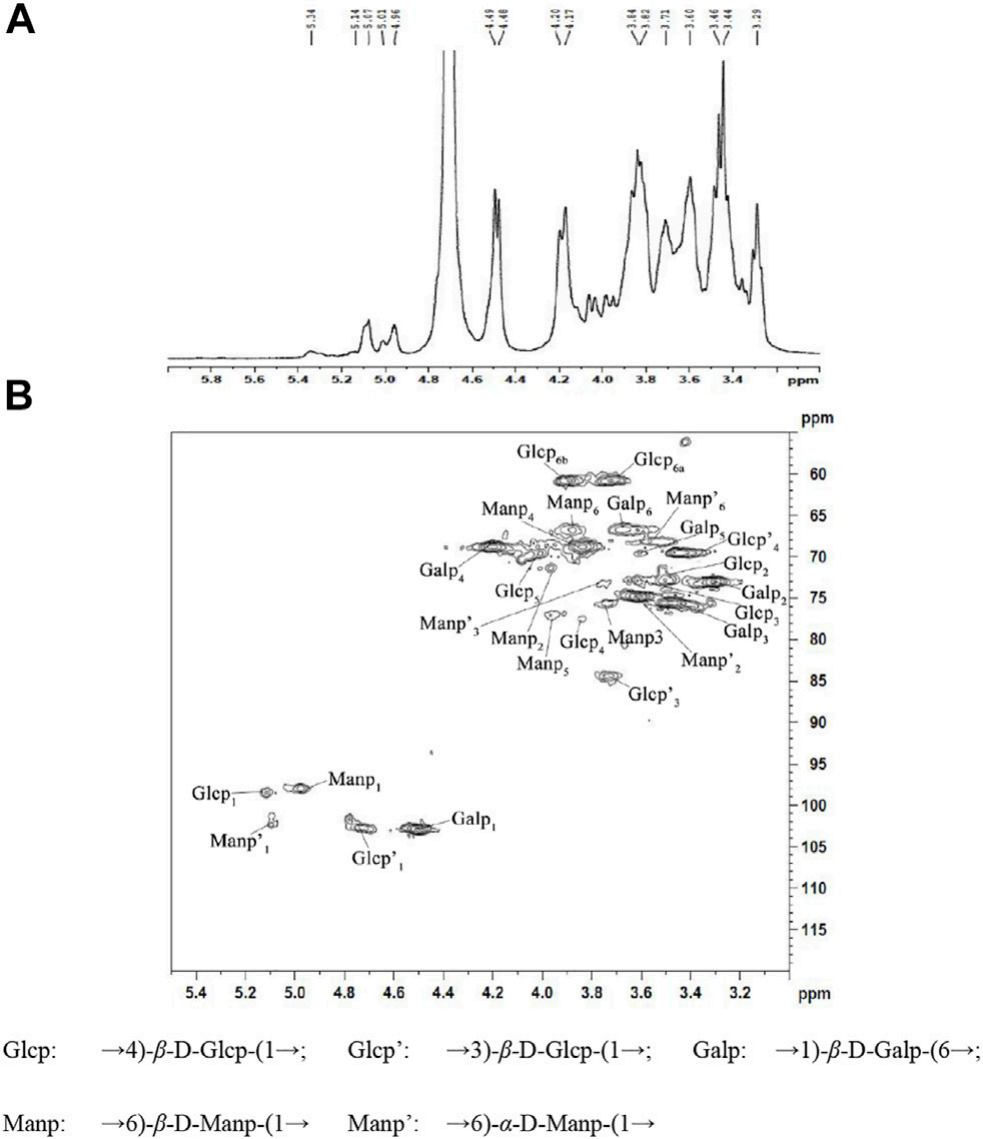
^1^H NMR spectra **(A)** (in D_2_O) and HSQC spectra **(B)** of NHSP.

1-D NMR spectra (^1^H) and 2-D NMR spectra (HSQC) of NHSP were obtained to further clarify structural features. In the ^1^H NMR spectrum of NHSP (Figure 3A), there were 16 anomeric hydrogen signals at δ 4.3–5.9. The anomeric proton signals at 3.49–4.10 ppm suggested that the configuration of NHSP was β-glucan in abundance. The signals at 4.20, 4.48, 4.49, 4.7, and 4.96 ppm indicate the presence of a large number of β-anomeric configuration residues, consistent with the presence of the 890 cm^−1^ peak on the FT-IR spectra. The weak signals at δ 5.01, δ 5.07, δ 5.14, and 5.34 were attributed to the anomeric α-pyranose configuration (Li et al., 2020; Liu et al., 2020; Yang et al., 2020).

In the HSQC spectrum of NHSP (Figure 3B), six cross-peaks of 5.12/98.5, 3.51/73.0, 3.62/73, 3.84/77.8, 4.01/70.1, and 3.74, 3.80/6 1.0 ppm were assigned to H-1/C-1, H-2/C-2, H-3/C-3, H-4/C-4, H-5/C-5 and H-6(a), and H-6(b)/C-6 of the →4) β-D-Glcp-(1→ residue, respectively. 1H/13C chemical shifts at 4.98/97.9, 3.96/71.98, 3.76/75.6, 3.85/69, 3.95/77, and 3.89/67.2 ppm were assigned to H-1/C-1, H-2/C-2, H-3/C-3, H-4/C-4, H-5/C-5, and H-6/C-6 of →6)-β-D-Manp-(1→ units, respectively, and cross peaks at 4.49/103, 3.32/73.1, 3.48/75.5, 4.21/69, 3.61/69.6, and 3.69/67 were assigned to H-1/C-1, H-2/C-2, H-3/C-3, H-4/C-4, H-5/C-5, and H-6/C-6 of →1)-β-D-Galp-(6→ units, respectively. The cross peaks at 4.75/102.7, 3.74/84.0, and 3.44/69.9 ppm were assigned to H-1/C-1, H-3/C-3, and H-4/C-4 of →3)-β-D-Glcp-(1→, respectively, while those at 5.10/102.8, 3.71/74.8, 3.79/73.6, and 3.76/68.2 corresponded to H-1/C-1, H-2/C-2, H-3/C-3, and H-6/C-6 of →6)-α-D-Manp-(1→ residues existing in NHSP (Kono et al., 2017; He et al., 2020; Li et al., 2020; Liu et al., 2020; Yang et al., 2020). These results agree with the FT-IR and ^1^H NMR results.

### Thermal Analysis

**FIGURE 4:**
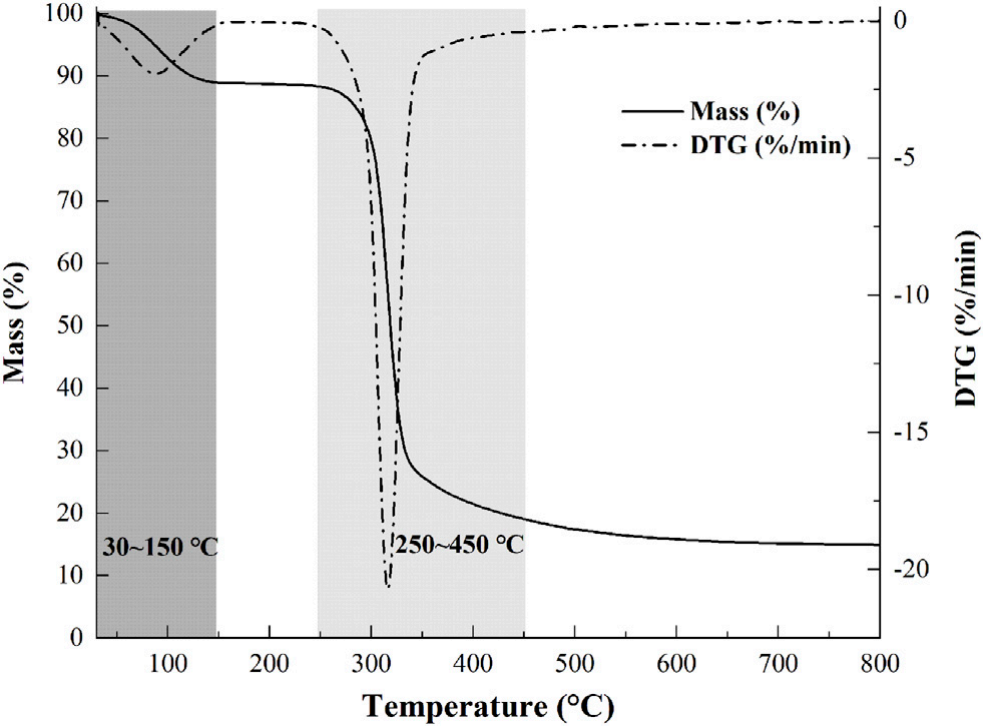
Weight loss percentage and derivative thermogravimetric curves of NHSP.

Thermostability is an important parameter for further applications of polysaccharides. The weight loss (TG) curve and derivative thermogravimetric (DTG) curve of the sample are presented in Figure 4. The process of mass loss of samples could be divided into two stages. A weight loss (∼10%) was observed from 30–150°C, which was attributed to the vaporization and removal of bound water in NHSP, which indicated the characteristic of moisture sorption for NHSP induced by the abundance of hydroxyl radical. Then, with the change of functional groups and the depolymerization of structure fractions, there occurred a significant loss (∼68%) of NHSP weight, indicating a severe decomposition within a temperature range (250–450°C). NHSP showed an onset degradation temperature (T_0_) at 282°C and a maximum degradation temperature (T_max_) at 316°C.

### Microstructure Analysis

**FIGURE 5:**
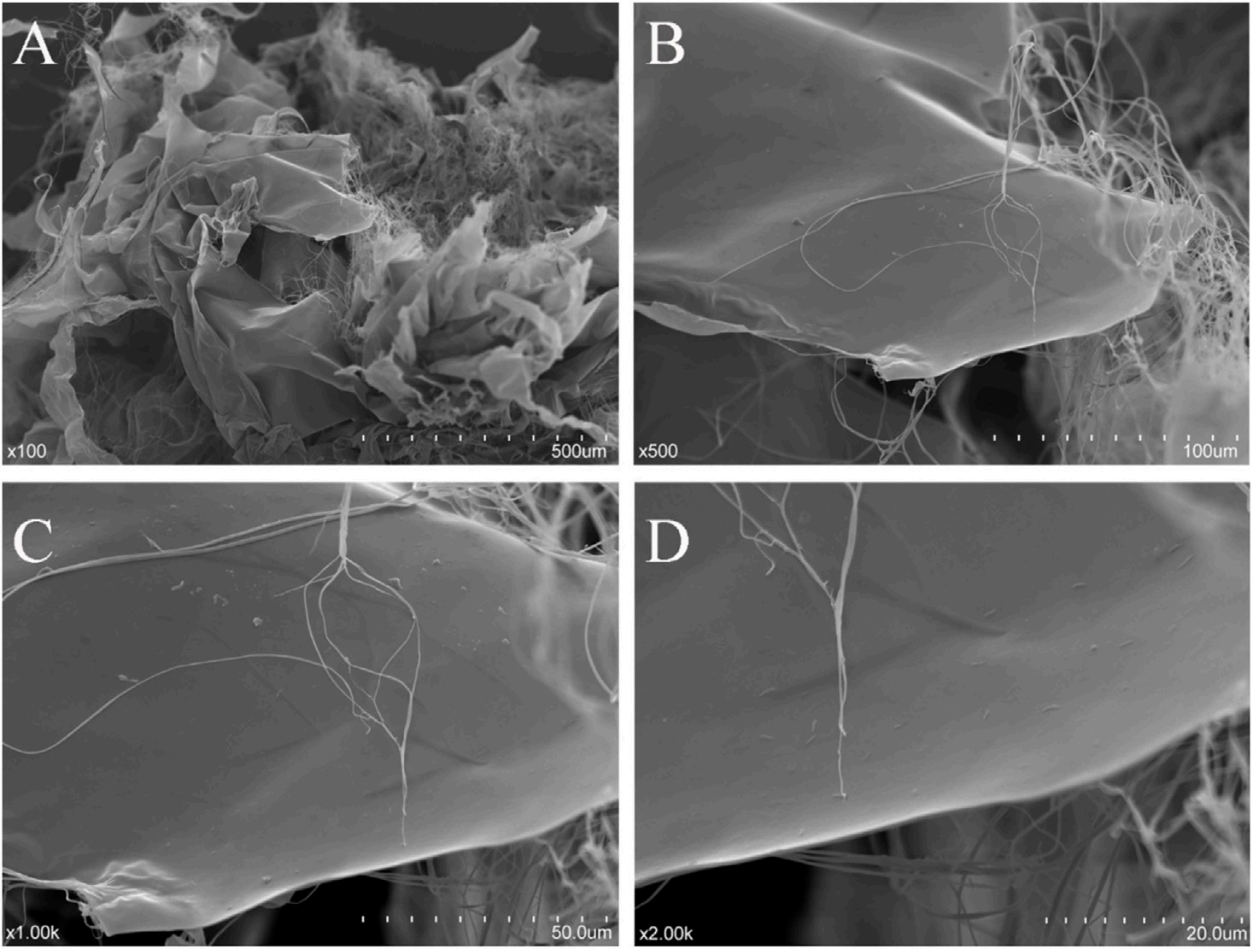
Scanning electron micrographs of NHSP [**(A)**: ×100; **(B)** ×500; **(C)** ×1,000; and **(D)** ×2000].

The microstructure of dialyzed NHSP is shown in Figure 5. Twisted ribbons with a smooth surface and some fibrous filaments existed in the purified NHSP, which might result in high viscosity (Zhou and Kang, 2018).

### NHSP Reduced FBG Without Effects on Body Weight in Diabetic Mice

**FIGURE 6:**
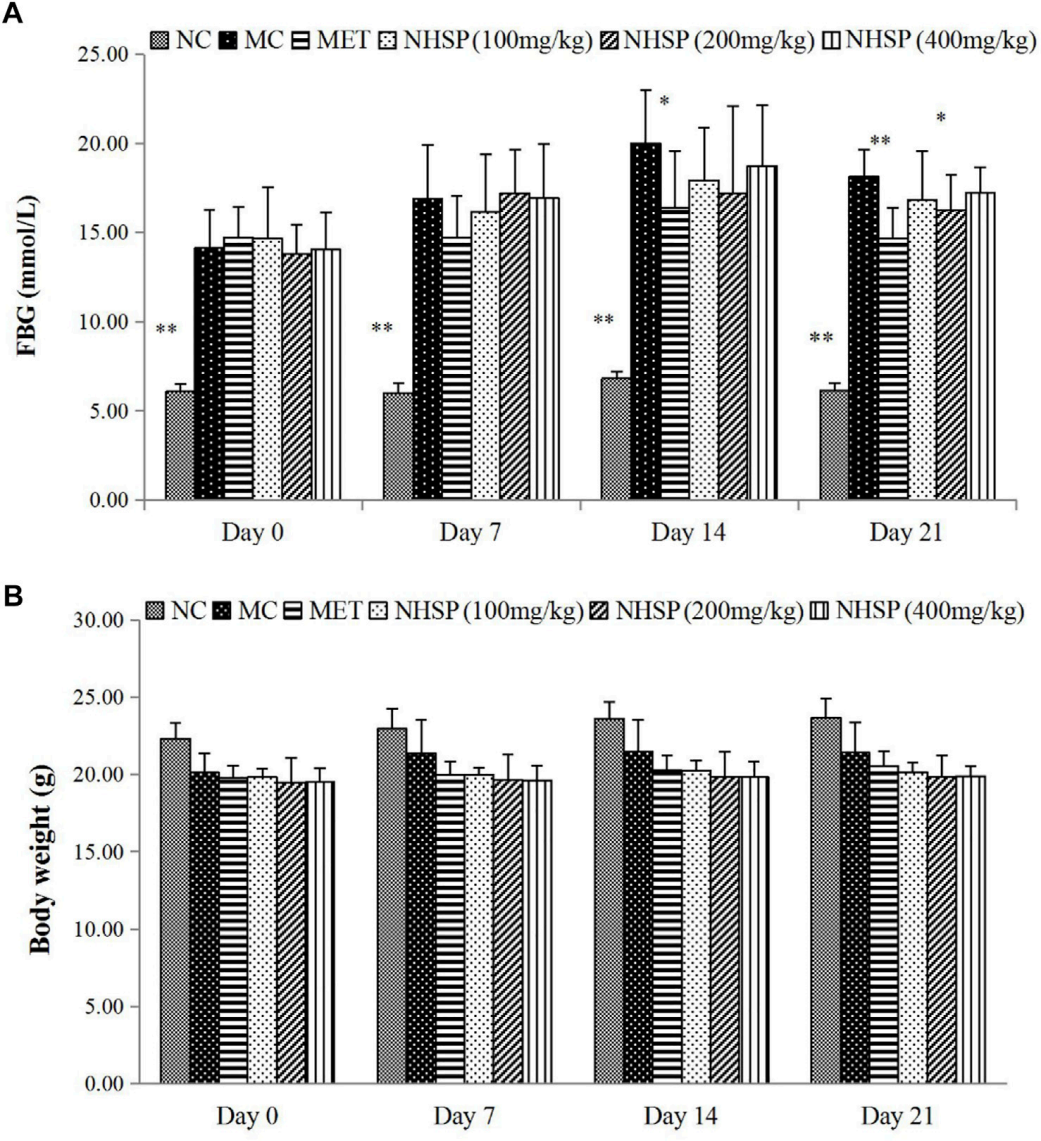
Effects of NHSP treatment on a type 2 diabetic mouse model. **(A)** Effect of NHSP on weight. **(B)** Effect of NHSP on FBG. Values are presented as mean ± S.D. *p < 0.05 for comparison with MC group. **p < 0.01 for comparison with MC group.

NHSP can lower the FBG levels of the type 2 diabetic mice model in all model groups (groups NHSP 100, NHSP 200, and NHSP 400) after 14 days but could not reach the effect of MET (group MET), and could not recover the FBG levels of normal mice (Group NC) (Figure 6A). Moreover, there was no dose-dependent effect of NHSP for FBG lowering in the type 2 diabetic mice model groups, and implied NHSP might reach its best effect in FBG controlling at some optimal dose range for NHSP intake. In another way, a relatively long time (>14 days) was needed for NHSP to lower FBG levels in the type 2 diabetic mice model, which indicated NHSP lowering FBG *in vivo* was not an immediate action mode. However, NHSP did not change the body weights for all groups significantly compared with groups NC, MC, and MET in 3 weeks (Figure 6B), which indicated that NHSP could be useful for the weight controlling of type 2 diabetic patients.

Moreover, the liver index was significantly (*p* < 0.05) lower in NHSP groups (groups NHSP 100, NHSP 200, and NHSP 400) than in NC, MC, and MET groups; but kidney, pancreas, or spleen indices had no evident changes (Table 2).

**TABLE 2:** Effects of NHSP on visceral indices.

Group	Dose (mg/kg)	Liver index (%)	Kidney index (%)	Spleen index (%)	Pancreas index (%)
NC	—	46.17 ± 8.48	13.08 ± 1.53	3.31 ± 0.50	7.97 ± 1.63*
MC	—	44.57 ± 2.89	12.95 ± 0.82	2.71 ± 0.86	6.29 ± 1.30
MET	200	45.54 ± 2.47	13.39 ± 1.47	3.31 ± 0.91	8.33 ± 1.50*
NHSP	100	40.48 ± 4.11*	12.97 ± 0.73	2.66 ± 0.85	6.55 ± 1.15
NHSP	200	41.11 ± 3.18*	13.36 ± 1.71	2.58 ± 0.31	6.62 ± 0.97
NHSP	400	40.95 ± 3.32*	12.32 ± 0.35	2.45 ± 0.23	7.36 ± 0.93

Values are presented as mean ± S.D. *p < 0.05 for comparison with the MC group.

## Discussion

*H. serotina* has many bioactivities to protect human health, but few works were done to evaluate the relationships between the hypoglycemic effect and its structures based on the pure *H. serotina* polysaccharides. In this work, the pure neutral polysaccharides from *H. serotina* fruiting bodies were purified and characterized, and their hypoglycemic effect was evaluated in the type 2 diabetic mouse model. This research suggested that neutral *H. serotina* polysaccharides (NHSP) can be a blood sugar-adapting potential functional food based on their special structures of links among sugars.

To yield the NHSP with high quality, the scheme was designed based on its characters with little electric charge. Here, the NHSP was purified with anion- and cation-chromatography consecutively, the unabsorbed fraction was eluted in one system at the same time, and collected at the final (Figure 1). The purification was completed at a low cost of time and the high purity polysaccharides were based on the characters with little electric charge.

Compared with previous research, the composition of crude HSP were glucose, galactose, mannose, ribose, and arabinose in a molar ratio of 14.24: 5.47: 9.35: 0.65: 0.69 (Li et al., 2013), and the purified HSPA1 with a small molecular weight of 0.809 kDa was composed of arabinose, mannose, glucose, and galactose in a molar ratio of 4: 16: 28: 11 (Li et al., 2017). NHSP with a similar composition to monosaccharides had been speculated have high bioactivity. NHSP was composed of glucose, galactose, and mannose in the molar ratio of 2.6: 2.1: 1.0. The composition of sugars in NHSP was simpler than that in HSP, and the proportion of glucose and galactose was higher. The neutral polysaccharide of *Armillaria mellea* had obvious hypoglycemic effects, and mannogalactoglycan was the main active area (Yang et al., 2019). The proportion of galactose in NHSP was higher, indicating that galactose played a major role. In general, mushroom polysaccharides with a molecular weight of around ×10^6^ kDa play the better role in inducing bioactivities. The results related to the composition of monosaccharides and the molecular weight in this experiment agreed with previous research.

Meantime, the high β-glycosidic bond content was the dominant structure in many mushroom polysaccharides that displayed high bioactivities, such as lentinan (from *Lentinula edodes*) based on its main chain consisting of β-(1→3)-D-glucan (Meng et al., 2016) and *Grifola frondosa* polysaccharides (He et al., 2017). The investigations *in vivo* for NHSP to control FBG indicated that the NHSP has hypoglycemic activity, coinciding with the high β-glycosidic bond content of NHSP. The result was similar to some polysaccharides with β-glycosidic bonds from other studies (Zhu et al., 2013; Liu et al., 2018).

Interestingly, type 2 diabetes and liver index are closely related (Firneisz, 2014). The liver index was relative to fat buildup in diabetes to some degree, which suggested that NHSP might help prevent hepatic steatosis or hepatomegaly associated with diabetes (Kasturiratne et al., 2013). In addition, NHSP can change the organ index. The neutral polysaccharides from *Hohenbuehelia serotina* can prevent apoptosis of splenocytes (Li et al., 2015). The spleen can activate T-lymphocytes and B-lymphocytes and play an important role in the body’s immune response.

## Conclusion

We characterized the neutral polysaccharides (NHSP) extracted from the mushroom *H. serotina*. NHSP was with higher total saccharide content (75.43 g/100 g) and lower protein content (1.98 g/100 g), and high homogeneity, indicated that the NHSP purification strategy was better. The monosaccharide component analysis showed that NHSP was composed of glucose, galactose, and mannose in molar ratio 2.6: 2.1: 1.0. FT-IR and NMR (^1^H and HSQC) spectroscopic analyses revealed that NHSP contained mainly 1,3-linked β-D-glucose, 1,4-linked β-D-glucose, 1,6-linked β-D-mannose, 1,6-linked α-D-mannose, and1,6-linked β-D-galactose. NHSP at a dose of 200 mg/kg for 21 days displayed hypoglycemic effects significantly in the type 2 diabetic mouse model, and NHSP could protect the liver by lowering the liver index. In summary, NHSP can play hypoglycemic and liver-protective roles based on its chemical structures, and this will provide a useful basis for further research on NHSP in its possible clinical applications.

## Data Availability

The original contributions presented in the study are included in the article/Supplementary Material, further inquiries can be directed to the corresponding author.

## References

[B1] Bao X. Yuan H. Wang C. Liu J. Lan M. (2013). Antitumor and Immunomodulatory Activities of a Polysaccharide from *Artemisia Argyi* . Carbohydr. Polym. 98 (1), 1236–1243. 10.1016/j.carbpol.2013.07.018 23987469

[B2] Bian J. Peng F. Peng X. P. Peng P. Xu F. Sun R. C. (2012). Acetic Acid Enhanced Purification of Crude Cellulose from Sugarcane Bagasse: Structural and Morphological Characterization. Bioresources 7 (4), 4626–4639. 10.15376/biores.7.4.4626-4639

[B3] Bin C. (2010). Optimization of Extraction of *Tremella Fuciformis* Polysaccharides and its Antioxidant and Antitumour Activities *In Vitro* . Carbohydr. Polym. 81 (2), 420–424.

[B4] Bradford M. M. (1976). A Rapid and Sensitive Method for the Quantitation of Microgram Quantities of Protein Utilizing the Principle of Protein-Dye Binding. Anal. Biochem. 72, 248–254. 10.1006/abio.1976.9999 942051

[B5] Cui L. Wang W. Luo Y. Ning Q. Xia Z. Chen J. (2019). Polysaccharide from Scutellaria Baicalensis Georgi Ameliorates Colitis via Suppressing NF-Κb Signaling and NLRP3 Inflammasome Activation. Int. J. Biol. Macromol 132, 393–405. 10.1016/j.ijbiomac.2019.03.230 30936012

[B6] Dubois M. Gilles K. A. Hamilton J. K. Rebers P. A. Smith F. (1956). Colorimetric Method for Determination of Sugars and Related Substances. Anal. Chem. 28 (3), 350–356. 10.1021/ac60111a017

[B7] Firneisz G. (2014). Non-alcoholic Fatty Liver Disease and Type 2 Diabetes Mellitus: the Liver Disease of Our Age? World J. Gastroenterol. 20 (27), 9072–9089. 10.3748/wjg.v20.i27.9072 25083080 PMC4112878

[B8] Guo C. Liang T. He Q. Wei P. Zheng N. Xu L. (2013). Renoprotective Effect of Ramulus Mori Polysaccharides on Renal Injury in STZ-Diabetic Mice. Int. J. Biol. Macromol 62, 720–725. 10.1016/j.ijbiomac.2013.09.022 24076200

[B9] Guo W. L. Chen M. Pan W. L. Zhang Q. Xu J. X. Lin Y. C. (2020). Hypoglycemic and Hypolipidemic Mechanism of Organic Chromium Derived from Chelation of *Grifola Frondosa* Polysaccharide-Chromium (III) and its Modulation of Intestinal Microflora in High Fat-Diet and STZ-Induced Diabetic Mice. Int. J. Biol. Macromol 145, 1208–1218. 10.1016/j.ijbiomac.2019.09.206 31726162

[B10] Guo W. L. Deng J. C. Pan Y. Y. Xu J. X. Hong J. L. Shi F. F. (2020). Hypoglycemic and Hypolipidemic Activities of *Grifola Frondosa* Polysaccharides and Their Relationships with the Modulation of Intestinal Microflora in Diabetic Mice Induced by High-Fat Diet and Streptozotocin. Int. J. Biol. Macromol 153, 1231–1240. 10.1016/j.ijbiomac.2019.10.253 31759027

[B11] Harsch I. A. Kaestner R. H. Konturek P. C. (2018). Hypoglycemic Side Effects of Sulfonylureas and Repaglinide in Ageing Patients - Knowledge and Self-Management. J. Physiol. Pharmacol. 69 (4), 647–649. 10.26402/jpp.2018.4.15 30552308

[B12] He B. L. Zheng Q. W. Guo L. Q. Huang J. Y. Yun F. Huang S. S. (2020). Structural Characterization and Immune-Enhancing Activity of a Novel High-Molecular-Weight Polysaccharide from Cordyceps Militaris. Int. J. Biol. Macromolinternational J. Biol. Macromolecules 145, 11–20. 10.1016/j.ijbiomac.2019.12.115 31846656

[B13] He X. Wang X. Fang J. Chang Y. Ning N. Guo H. (2017). Polysaccharides in *Grifola Frondosa* Mushroom and Their Health Promoting Properties: A Review. Int. J. Biol. Macromol 101, 910–921. 10.1016/j.ijbiomac.2017.03.177 28366857

[B14] Hwang H. J. Kim S. W. Lim J. M. Joo J. H. Kim H. O. Kim H. M. (2005). Hypoglycemic Effect of Crude Exopolysaccharides Produced by a Medicinal Mushroom *Phellinus Baumii* in Streptozotocin-Induced Diabetic Rats. Life Sci. 76 (26), 3069–3080. 10.1016/j.lfs.2004.12.019 15850599

[B15] Kasturiratne A. Weerasinghe S. Dassanayake A. S. Rajindrajith S. de Silva A. P. Kato N. (2013). Influence of Non-alcoholic Fatty Liver Disease on the Development of Diabetes Mellitus. J. Gastroenterol. Hepatol. 28 (1), 142–147. 10.1111/j.1440-1746.2012.07264.x 22989165

[B16] Kono H. Kondo N. Hirabayashi K. Ogata M. Totani K. Ikematsu S. (2017). NMR Spectroscopic Structural Characterization of a Water-Soluble β-(1 → 3, 1 → 6)-glucan from Aureobasidium Pullulans. Carbohydr. Polym. 174, 876–886. 10.1016/j.carbpol.2017.07.018 28821143

[B17] Li L. Xu J. X. Cao Y. J. Lin Y. C. Guo W. L. Liu J. Y. (2019). Preparation of Ganoderma Lucidum Polysaccharide-chromium (III) C-omplex and its H-ypoglycemic and H-ypolipidemic A-ctivities in H-igh-F-at and H-igh-F-ructose D-iet-I-nduced P-re-diabetic M-ice. Int. J. Biol. Macromol 140, 782–793. 10.1016/j.ijbiomac.2019.08.072 31401268

[B18] Li M.-F. Fan Y.-M. Xu F. Sun R.-C. (2011). Structure and thermal Stability of Polysaccharide Fractions Extracted from the Ultrasonic Irradiated and Cold Alkali Pretreated Bamboo. J. Appl. Polym. Sci. 121 (1), 176–185. 10.1002/app.33491

[B19] Li X. Wang L. Wang Z. (2015). Radioprotective Activity of Neutral Polysaccharides Isolated from the Fruiting Bodies of *Hohenbuehelia Serotina* . Phys. Med. 31 (4), 352–359. 10.1016/j.ejmp.2015.02.004 25703009

[B20] Li X. Wang L. Wang Z. (2017). Structural Characterization and Antioxidant Activity of Polysaccharide from *Hohenbuehelia Serotina* . Int. J. Biol. Macromol 98, 59–66. 10.1016/j.ijbiomac.2016.12.089 28069348

[B21] Li X. Wang Z. Wang L. (2013). Polysaccharide of *Hohenbuehelia Serotina* as a Defense against Damage by Whole-Body Gamma Irradiation of Mice. Carbohydr. Polym. 94 (2), 829–835. 10.1016/j.carbpol.2013.02.015 23544639

[B22] Li Y. You L. Dong F. Yao W. Chen J. (2020). Structural Characterization, Antiproliferative and Immunoregulatory Activities of a Polysaccharide from Boletus *Leccinum Rugosiceps* . Int. J. Biol. Macromol 157, 106–118. 10.1016/j.ijbiomac.2020.03.250 32289425

[B23] Liu W. Lv X. Huang W. Yao W. Gao X. (2018). Characterization and Hypoglycemic Effect of a Neutral Polysaccharide Extracted from the Residue of *Codonopsis Pilosula* . Carbohydr. Polym. 197, 215–226. 10.1016/j.carbpol.2018.05.067 30007607

[B24] Liu X. Liu D. Chen Y. Zhong R. Gao L. Yang C. (2020). Physicochemical Characterization of a Polysaccharide from *Agrocybe Aegirita* and its Anti-ageing Activity. Carbohydr. Polym. 236, 116056. 10.1016/j.carbpol.2020.116056 32172871

[B25] Long A. N. Dagogo-Jack S. (2011). Comorbidities of Diabetes and Hypertension: Mechanisms and Approach to Target Organ protection. J. Clin. Hypertens. (Greenwich) 13 (4), 244–251. 10.1111/j.1751-7176.2011.00434.x 21466619 PMC3746062

[B26] Meng X. Liang H. Luo L. (2016). Antitumor Polysaccharides from Mushrooms: a Review on the Structural Characteristics, Antitumor Mechanisms and Immunomodulating Activities. Carbohydr. Res. 424, 30–41. 10.1016/j.carres.2016.02.008 26974354

[B27] Moradali M. F. Mostafavi H. Ghods S. Hedjaroude G. A. (2007). Immunomodulating and Anticancer Agents in the Realm of Macromycetes Fungi (Macrofungi). Int. Immunopharmacol 7 (6), 701–724. 10.1016/j.intimp.2007.01.008 17466905

[B28] Peng F. Ren J. L. Xu F. Bian J. Peng P. Sun R. C. (2010). Fractionation of Alkali-Solubilized Hemicelluloses from Delignified *Populus Gansuensis*: Structure and Properties. J. Agric. Food Chem. 58 (9), 5743–5750. 10.1021/jf1003368 20302372

[B29] Radzki W. Ziaja-Sołtys M. Nowak J. Rzymowska J. Topolska J. Sławińska A. (2016). Effect of Processing on the Content and Biological Activity of Polysaccharides from *Pleurotus Ostreatus* Mushroom. LWT - Food Sci. Technol. 66, 27–33. 10.1016/j.lwt.2015.10.016

[B30] Rozi P. Abuduwaili A. Mutailifu P. Gao Y. Rakhmanberdieva R. Aisa H. A. (2019). Sequential Extraction, Characterization and Antioxidant Activity of Polysaccharides from *Fritillaria Pallidiflora* Schrenk. Int. J. Biol. Macromol 131, 97–106. 10.1016/j.ijbiomac.2019.03.029 30844453

[B31] Sun Y. Wang S. Li T. Li X. Jiao L. Zhang L. (2008). Purification, Structure and Immunobiological Activity of a New Water-Soluble Polysaccharide from the Mycelium of *Polyporus Albicans* (Imaz.) Teng. Bioresour. Technol. 99 (4), 900–904. 10.1016/j.biortech.2007.01.029 17368889

[B32] Talchai C. Xuan S. Lin H. V. Sussel L. Accili D. (2012). Pancreatic β Cell Dedifferentiation as a Mechanism of Diabetic β Cell Failure. Cell 150 (6), 1223–1234. 10.1016/j.cell.2012.07.029 22980982 PMC3445031

[B33] Tfayli H. Bacha F. Gungor N. Arslanian S. (2009). Phenotypic Type 2 Diabetes in Obese Youth: Insulin Sensitivity and Secretion in Islet Cell Antibody-Negative versus -positive Patients. Diabetes 58 (3), 738–744. 10.2337/db08-1372 19073767 PMC2646074

[B34] Wu J. Chen M. Shi S. Wang H. Li N. Su J. (2017). Hypoglycemic Effect and Mechanism of a Pectic Polysaccharide with Hexenuronic Acid from the Fruits of Ficus Pumila L. In C57BL/KsJ Db/db Mice. Carbohydr. Polym. 178, 209–220. 10.1016/j.carbpol.2017.09.050 29050587

[B35] Xiao C. Wu Q. P. Cai W. Tan J. B. Yang X. B. Zhang J. M. (2012). Hypoglycemic Effects of *Ganoderma Lucidum* Polysaccharides in Type 2 Diabetic Mice. Arch. Pharm. Res. 35 (10), 1793–1801. 10.1007/s12272-012-1012-z 23139131

[B36] Yang S. Yan J. Yang L. Meng Y. Wang N. He C. (2019). Alkali-soluble Polysaccharides from Mushroom Fruiting Bodies Improve Insulin Resistance. Int. J. Biol. Macromol 126, 466–474. 10.1016/j.ijbiomac.2018.12.251 30594618 PMC8593897

[B37] Yang X. Wu Y. Zhang C. Fu S. Zhang J. Fu C. (2020). Extraction, Structural Characterization, and Immunoregulatory Effect of a Polysaccharide Fraction from *Radix Aconiti Lateralis Preparata* (Fuzi). Int. J. Biol. Macromol 143, 314–324. 10.1016/j.ijbiomac.2019.11.208 31786293

[B38] Yuan Z. He P. Cui J. Takeuchi H. (1998). Hypoglycemic Effect of Water-Soluble Polysaccharide from *Auricularia Auricula-judae* Quel. On Genetically Diabetic KK-Ay Mice. Biosci. Biotechnol. Biochem. 62 (10), 1898–1903. 10.1271/bbb.62.1898 9836425

[B39] Zhang J. Yu Y. Zhang Z. Ding Y. Dai X. Li Y. (2011). Effect of Polysaccharide from Cultured Cordyceps Sinensis on Immune Function and Anti-oxidation Activity of Mice Exposed to 60Co. Int. Immunopharmacol 11 (12), 2251–2257. doi: 10.1016/j.intimp.2011.09.019 22001898

[B40] Zhang M. Cui S. W. Cheung P. C. K. Wang Q. (2006). Polysaccharides from Mushrooms: a Review on Their Isolation Process, Structural Characteristics and Antitumor Activity. Trends Food Sci. Technol. 18 (1), 4–19. 10.1016/j.tifs.2006.07.013

[B41] Zhang Z. Wang X. Zhao M. Qi H. (2014). Free-radical Degradation by Fe2+/Vc/H2O2 and Antioxidant Activity of Polysaccharide from Tremella Fuciformis. Carbohydr. Polym. 112, 578–582. 10.1016/j.carbpol.2014.06.030 25129784

[B42] Zhou R. Kang Y. H. (2018). Synergistic Interaction of *Auricularia Auricula-judae* Polysaccharide with Yam Starch: Effects on Physicochemical Properties and *In Vitro* Starch Digestibility. Food Sci. Biotechnol. 27 (6), 1579–1588. 10.1007/s10068-018-0419-9 30483421 PMC6233405

[B43] Zhu J. Liu W. Yu J. Zou S. Wang J. Yao W. (2013). Characterization and Hypoglycemic Effect of a Polysaccharide Extracted from the Fruit of *Lycium Barbarum* L. Carbohydr. Polym. 98 (1), 8–16. 10.1016/j.carbpol.2013.04.057 23987311

